# The Influence Research on Nitrogen Transport and Reaction in the Hyporheic Zone with an In-Stream Structure

**DOI:** 10.3390/ijerph191912695

**Published:** 2022-10-04

**Authors:** Ruikang Sun, Jiawei Dong, Yi Li, Panwen Li, Yaning Liu, Ying Liu, Jinghong Feng

**Affiliations:** 1 Hubei Key Laboratory of Ecological Restoration of River-Lakes and Algal Utilization, Hubei University of Technology, Wuhan 430068, China; 2 Foreign Environmental Cooperation Center, Ministry of Ecology and Environment of China, Beijing 100035, China; 3 Department of Mathematical and Statistical Sciences, University of Colorado Denver, Denver, CO 80204, USA

**Keywords:** the hyporheic zone, in-stream weir structure, nitrogen, flume experiment, numerical simulation

## Abstract

The hyporheic zone (HZ) is important for river ecological restoration as the main zone with nitrogen biochemical processes. The engineering of river ecological restoration can significantly change the hydrodynamics, as well as solute transport and reaction processes, but it is still not fully understood. In this study, nitrogen transport and reaction processes were analyzed in the HZ with an in-stream weir structure. An HZ model was built, and three reactions were considered with different design parameters of the weir structure and different permeability characteristics of porous media. The results show that a structure with a greater height on the overlying surface water enables the species to break through deeper porous media. It promotes the mean spatial reaction rates of nitrification and denitrification and results in increased net denitrification in most cases. In addition, increasing the burial depth of the structure leads to the same variation trends in the mean spatial reaction rates as increasing the structure height. Larger permeability coefficients in porous media can enhance flow exchange and increase mean spatial reaction rates. The results can help deepen the understanding of nitrogen transport and transformation in the HZ and optimize the design parameters and location of the in-stream structure.

## 1. Introduction

Nitrogen pollution is an important environmental problem that rivers are facing, and it has become a critical objective for river ecological restoration [1]. It was found that more than half of the nitrogen entering the river can be finally converted into N_2_ emission by biochemical processes. The main biochemical nitrogen processes occur in the hyporheic zone (HZ), a saturated water area under or beside the riverbed with mixed shallow groundwater and surface water [2], which makes the entire physical, chemical, and biological processes more complex [3]. Research on the different influential processes of the HZ has been drawing a great deal of interest.

Transport and reactions of nitrogen or other species are mainly influenced by the distribution of some environmental factors, which are controlled by hyporheic exchanges [4,5]. The downwelling flow in the HZ is an important mechanism for carrying oxygen-rich surface water and pollutants into sediments and groundwater, which provides abundant dissolved oxygen and organic matter [6]. The upwelling flow in the HZ releases pore water containing low oxygen-reducing species back into the overlying surface water [7]. In addition, the water exchange path and residence time play an important role in the biochemical processes in the HZ [8]. In previous research, study methods including indoor flume, field experiments, and numerical simulation have been conducted to analyze the impact of natural factors, such as riverbed morphology, riverbank morphology, and sediment distribution, on the hyporheic exchange process [9,10,11,12]. Hester et al. used the coupled model of surface water and groundwater to analyze the factors including riverbed permeability characteristics, riverbed slope, and groundwater head for the nitrate removal in the HZ [13,14]. Zheng et al. found that the effect of temperature variation on the daily average nitrate removal efficiency is not obvious compared with steady-temperature systems [15,16]. Ping et al. used COMSOL to study the impact of natural riverbed fluctuation on nitrification and denitrification [17]. The results showed that large morphology fluctuation can accelerate the nitrogen reaction. Further, Ping et al. studied the development of the bioclogging effect on the nitrate source and sink function in HZ by a numerical model. In this research, they set a single triangular dune streambed and the ANSYSY Fluent was used to calculate the sediment–water interface pressure, and COMSOL was used for flow and reaction in porous media [18]. Liu et al. also adopted COMSOL to build a two-dimensional numerical model with a series of dunes for evaluating the nitrogen removal rate. A new model coupling the basic biochemical reaction and genetic programming was proposed to delineate the boundary between nitrification and denitrification [19]. Mendoza et al. analyzed the influence process of hydraulic conductivity in the hyporheic sediments on the nitrogen uptake from both the reach and hyporheic scale. It found that hydraulic conductivity is important for determining the contribution of hydrological exchange in different scales and thus influences nitrogen uptake [20]. Pescimoro et al. discussed the hyporheic exchange in physically and chemically heterogeneous sediments using the simulation method and found that a higher volume proportion of silt is a more efficient nitrate removal. In the paper, they computed the hydraulic head distribution of idealized channel geometry for the sediment–water interface first and then adopted MODFLOW to discuss the process in the HZ [21].

Focusing on the improvement of the river environment, more and more river ecological restoration projects have been established, which can change the natural conditions of surface water and sediment and will affect the hydrodynamics and solute migration process in the hyporheic zone [22,23,24,25]. Mutz et al. conducted an experiment to change the river channel morphology by adding wood. It was found that flow resistance can increase the vertical water flux through the riverbed by 1.8 to 2.5 times compared with structures with no wood [26]. Sawyer et al. verified that weir structure can improve the path length of the hyporheic exchange and residence time by a flume experiment [27]. Rana et al. constructed a sequence of weirs in the river to simulate the NaCl movement process with artificial structures, such as log dams, pebble dams, and debris dams [28]. The results showed that weirs can result in increases in both the surface water flow and the cross-sectional area of the transient storage area. Hester and Doyle analyzed the effect of different structures on hyporheic flow and showed that channel crossing structures are more prominent than partial crossing structures [13]. Li et al. conducted an indoor plume experiment and used a numerical model to study the NaCl solute transport in the HZ with an in-stream structure [29]. Non-reactive solute transport depth, hyporheic vertical exchange flux, and solute flux can increase with the ratio of ground height to the underground part and structure number. Ward et al. found that hyporheic flux can be impacted by the structure design parameters [30]. Liu and Chui built models with different weir heights to consider the impact on denitrification in the HZ. It indicated that a possible optimal weir height exists when some assumptions are met [31]. Tewari et al. summarized the current research development of the engineered hyporheic zone and discussed the limitations of the main three methods including the field experiment, flume experiment, and numerical models [32]. Moreover, numerical simulation has been an important study method from previous research on hydrodynamic and reaction processes in the HZ. The coupling of the surface water and flow in porous media with different governing equations is the key process. Generally, the sediment–water interface pressure is assumed as the connecting boundary, and the coupling process is computed by two simulation software or methods for surface water and groundwater, respectively, such as ANASYS Fluent and some empirical functions for surface water, MODFLOW, FEFLOW, and other software for porous media. In comparison with these separation forms, COMSOL can be used for both processes and is convenient for the coupled simulation, which has been adopted in some research [17,18,19,29,33].

Most previous studies focused on the hydrodynamic process and nitrogen migration and transformation under natural conditions. Relatively few studies considered the influence of ecological structure design on the entire nitrogen reaction process, and the research results cannot guide practical applications well and restrict the further optimization of ecological engineering. Moreover, in our previous research [29,33], we only limited our scope to analyzing the hydrodynamics process and the diffusion of conserved substances with a weir structure by the flume experiment and numerical simulation. The main factors controlling the hydrodynamic process and the diffusion of conserved substances have been well understood, but the effects on the more complex processes of nitrogen migration and transformation with the in-stream weir structure have not yet been discussed, which we considered our ultimate goal for river ecological restoration design. In this paper, the same indoor flume experiment as that used in our previous research was conducted and a hydrodynamic reaction HZ model with a weir structure was considered. The basic model was verified by the real data of the flume experiment through a NaCl solute. A hypothetical nitrogen transport and reaction model considering three main reaction processes and four species was established. It aimed to answer two fundamental questions. The first was how the special weir structure impacts the nitrogen transport and transformation in the HZ and how the main structure design parameters influence the process. Second, we discussed the impacts of the homogeneous permeability characteristic in porous media on nitrogen transport and transformation in the HZ. The research can help deepen the understanding of nitrogen transport and transformation with in-stream structures and optimize the design parameters.

## 2. Materials and Methods

### 2.1. Flume Experiment

**Figure 1:**
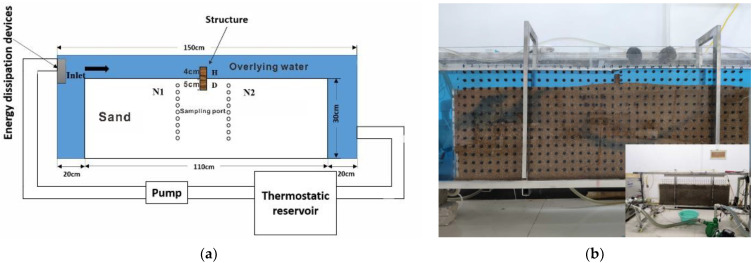
Schematic diagram of the flume experiment (**a**) revised from Refs. [11,33] and the actual experimental flume diagram (**b**). The light gray and blue areas in (**a**) represent the parts filled with sand and water, respectively. The object is the enlarged flume with weir and the embedded is the overall peripheral structure design in (**b**).

An indoor recirculating flume experiment was conducted to study the flow process in the hyporheic zone based on previous research [11,29,33]. As shown in Figure 1, the scale of the flume was set as 1.5 × 0.5 × 0.1 m. Acrylic acid (1 cm-thick) was used to build the tank wall to facilitate experimental observation. In order to avoid tumbling and fluctuation of the flow at the inlet, we installed an energy dissipation device at the inlet of the flume. The flow velocity of the overlying water was set to 0.04 m/s controlled by a valve and measured by an electromagnetic flowmeter. In addition, the experiment temperature is constantly controlled by an additional water recycling side loop. Several small holes at different depths for sampling are arranged on the side of the flume to avoid fluctuation during the sampling process.

In the experiment, sand or gravel with a particle size of 0.25 to 0.5 mm were used as the riverbed filler. The sand was rinsed three times with tap water to remove impurities and organic matter. In this device, the length and width of the sand layer as the riverbed are 1.1 and 0.3 m, respectively. A 9 cm-high channel-spanning weir with impermeable material was set at the center of the channel. The depth in the porous medium (burial depth) of the structure was 5 cm, and the height of the structure at the water interface was 4 cm. At the beginning of the experiment, the flume was filled with tap water, and the sand was laid in a saturated condition. The constant temperature was set to 20 °C by a water tank with a constant temperature. NaCl was selected as the non-reactive tracer to study the flow condition in the research. NaCl dissolved completely and slowly added to the recirculating water tank, to ensure that the initial concentration in the tank was 2.3 g/L. In order to monitor the process of non-reactive solute transport in the porous medium, pore water samples were taken over time from the samples’ ports. The volume of pore water samples was 100 uL, which could not significantly affect the flow field variations near the sampling ports. The water samples were diluted with 5 ml of deionized water to guarantee enough volume for an electrical conductivity (EC) measurement with the device of the Swiss-made Mettler Toledo S230. It is convenient to obtain the NaCl concentration distribution because the relationship between the concentration and EC of NaCl is linear, especially in a low concentration range.

### 2.2. Numerical Simulation

#### 2.2.1. Conceptual Coupled Model

**Figure 2:**
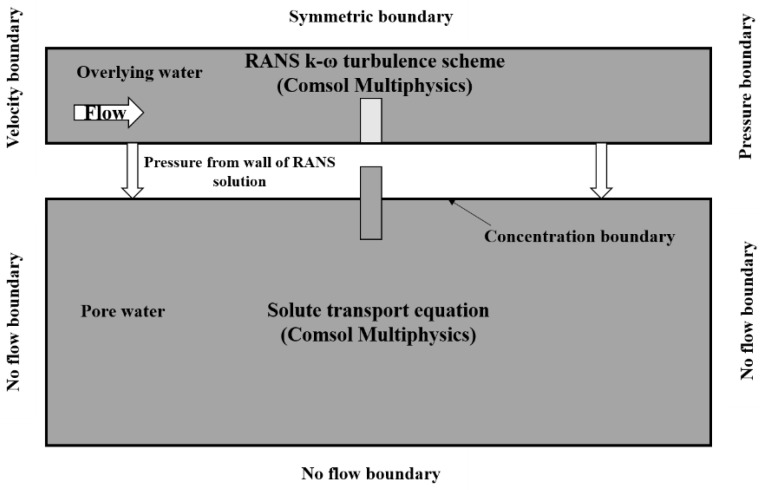
The conceptual model for the hyporheic zone in the experiment revised from Refs. [11,33].

As mentioned above, the hyporheic zone is complex and controlled by both the surface flow domain and groundwater flow domain. In this study, the coupled modeling scheme follows previous studies [16,29,34] and the conceptual model is shown in Figure 2. The pressure distribution of the sediment–water (SWI) was used as the pressure boundary for the groundwater (pore water) domain. It can be simulated by COMSOL using the mean unidirectional turbulent flow by solving the Reynolds-averaged Navier–Stokes (RANS) equations and the k-ω turbulence model. A symmetric boundary was adopted on the top of the overlying water, and the constant velocity and pressure conditions were set at the left and right boundaries, respectively. In the overlying domain, we considered that the entire ecological weir was always below the water surface, and the unstable falling process through the weir with low water was ignored, which was the same condition as the flume experiment. The overlying water stable flow process was simulated to ensure that the SWI pressure distribution was unchanged under the same weir condition. The solute transport and reaction mainly occurred in the pore water domain and the transport and reaction model of groundwater was used. According to the basic conditions of the experiment, some assumptions were made including (1) that the flow was steady and incompressible, and (2) that the sand in the bed was without displacement, homogeneous, and isotropic. In the pore domain, all boundaries were set to no flow boundaries, except for the upper-pressure boundary.

#### 2.2.2. Hydrodynamic Mathematical Model

The flow was governed by the Reynolds-averaged Navier–Stokes (RANS) equations and the k-ω turbulence model [10]. For an incompressible fluid, the steady state RANS equations are defined as:

(1) $$\frac{\partial \rho U_{i}}{x_{i}}=0$$

(2) $$\rho U_{j}\frac{\partial U_{j}}{\partial x_{i}}=-\frac{\partial p}{\partial x_{i}}+\frac{\partial }{\partial x_{j}}(2\mu S_{i,j}-\rho \bar{u_{j}^{\prime }u_{i}^{\prime }})$$

where ρ, μ, and *p* refer to fluid density, dynamic viscosity, and average pressure respectively, $U_{i}$ or $U_{j}$ (*i*, *j* = 1, 2, where *i* ≠ *j*) is the time-averaged velocity, and $u_{i}^{\prime }$ or $u_{j}^{\prime }$ refers to the fluctuation in the instantaneous velocity component in the $x_{i}$ or $x_{j}$ (*i*, *j* = 1, 2, where i ≠ j) direction. The strain rate tensor ($S_{i,j}$) is defined as:

(3) $$S_{i,j}=\frac{1}{2}(\frac{\partial U_{i}}{\partial x_{j}}+\frac{\partial U_{j}}{\partial x_{i}})$$

The Reynolds stresses are related to turbulent kinetic energy (k) and specific dissipation rate ω by:

(4) $$-\bar{u_{j}^{\prime }u_{i}^{\prime }}=v_{t}(2S_{i,j})-\frac{2}{3}\delta_{ij}k$$

where $v_{t}$ refers to kinematic eddy viscosity, and $\delta_{ij}$ refers to the Kronecker delta.

The eddy viscosity in this closure scheme is:

(5) $$v_{t}=\frac{k}{\omega }$$

The expression of the specific dissipation coefficient is:

(6) $$\omega =\frac{\varepsilon }{\beta^{*}k}$$

where $\beta^{*}$ refers to the closure coefficient and ε refers to the turbulent dissipation rate.

The k equation and ω equation of the k-ω turbulence model are, respectively:

(7) $$\rho \frac{\partial (U_{j}k)}{\partial x_{j}}=\rho \tau_{ij}\frac{\partial U_{i}}{\partial x_{j}}-\beta^{*}\rho \omega k+\frac{\partial }{\partial x_{j}}\left[(u+\rho v_{t}\sigma_{k})\frac{\partial k}{\partial x_{j}}\right]$$

(8) $$\rho \frac{\partial (U_{j}\omega )}{\partial x_{j}}=\alpha \frac{\rho \omega }{k}\tau_{ij}\frac{\partial U_{i}}{\partial x_{j}}-\beta \rho \omega^{2}+\frac{\partial }{\partial x_{j}}\left[(u+\rho v_{t}\sigma_{\omega })\frac{\partial \omega }{\partial x_{j}}\right]$$

The standard closure coefficients for the k-ω scheme are: α = 5/9, β = 3/40, $\beta^{*}\;$= 9/100, and $\sigma_{k\;}$=$\;\sigma_{\omega }\;$= 1/2.

Darcy’s Law for groundwater is:

(9) $$u=-\frac{\kappa }{\mu }(\nabla p+\rho g\nabla D)$$

where u is the Darcy velocity (m/s), κ is the permeability coefficient (m/s), ∇D is the vector unit in the direction of gravity, and g is the magnitude of gravitational acceleration.

The time-dependent non-reactive solute transport is modeled as:

(10) $$\frac{\partial C}{\partial t}=D\frac{\partial^{2}C}{\partial x_{i}^{2}}-\frac{q_{i}}{\theta }\frac{\partial C}{\partial x_{i}}$$

where θ is the porosity, D is the dynamic dispersion tensor of fluid, C is the molar concentration of NaCl, and $q_{i}$ is the Darcy flux in the corresponding direction.

#### 2.2.3. Nitrogen Reactive Transport Modeling

As our research objective, a hypothetical nitrogen transport and reaction model was run after the non-reactive model was validated by the experiment data. In this study, four representative reactive compounds were selected for nitrogen-related reactions: dissolved oxygen (DO), dissolved organic carbon (DOC), nitrate (NO_3_^−^), and ammonium ion (NH_4_^+^) [34]. Because of its simple chemical structure, formaldehyde (CH_2_O) was used to represent DOC in the study. Particulate organic carbon (POC) was not considered in this study because POC particles affect sediment permeability and microbial distribution, which may complicate the modeling of nitrogen migration and transformation. It was assumed that all nutrients in the sediment were percolated by the flow of water in the riverbed. Three key chemical reactions of nitrogen biogeochemical reactions were selected in the multi-component reactive migration model: nitrification (NI), denitrification (DN), and aerobic respiration (AR) of dissolved organic matter. The detailed reaction equations used in the model are illustrated in Table 1. Other reactions of nitrogen were ignored.

The steady-state reactive nitrogen transport equation in porous media can be described as:

(11) $$\nabla (-\theta D\nabla C_{j}+qC_{j})=\theta R_{j}$$

where $R_{j}$ is the net reaction rate of the four substances, and $C_{j}$ is the molar concentration of the four species. The dynamic dispersion tensor of fluid is calculated by:

(12) $$\theta D=\alpha_{T}\left|q\right|\delta_{ij}+(\alpha_{L}-\alpha_{T})\frac{q_{i}q_{j}}{\left|q\right|}+\theta \tau D_{m}\delta_{ij}$$

where $\alpha_{L}$ is the lateral dispersion, $\alpha_{T}$ refers to the longitudinal dispersion, $\alpha_{L}$/$\alpha_{T}\;$= 10, τ is the tortuosity factor, $\delta_{ij}$ (*i*,*j* = 1,2) is the Kronecker delta function, and $D_{m}$ is the molecular diffusivity in porous media.

In this study, the degradation of organic compounds was considered the ultimate source of chemical energy. For simplicity, the DOC oxidation rate $r_{\mathrm{DOC}}$ was assumed to be the first-order degradation kinetics:

(13) $$r_{DOC}=k_{DOC}\cdot C_{DOC}$$

where $k_{\mathrm{DOC}}$ is the first-order rate constant, and $C_{\mathrm{DOC}}$ is the molar concentration of *DOC*. The linear dynamics of this equation was the simplest way to express *DOC* degradation [35]. Aerobic respiration and nitrification were assumed to occur simultaneously, while denitrification occurred only when oxygen concentration dropped below the limit value. It is assumed that the electrons generated by *DOC* degradation are transferred to potential terminal electron acceptors, which in turn are utilized by microorganisms:

(14) $$r_{red,i}=f_{i}\cdot r_{DOC}\cdot \beta_{i}(i=1,2)$$

where $f_{i}$ is the number of electrons consumed by the *i*-th reduction half-reaction, and $\beta_{i}$ is the ratio of the number of transferred electrons per mole of oxidized *DOC* to the number of electrons per mole of the reduced compound in the *i*-th reaction. The component $f_{i}$ is defined using the simplified Monod formula of the terminal electron acceptor [36]:

(15) $$f_{i}=(1-\sum_{n=0}^{i-1}f_{n})\cdot \alpha_{i}$$

(16) $$\alpha_{i}=\{\begin{array}{c} \frac{C_{i}}{C_{i,\lim}}\to if(C_{i}<C_{i,\lim})\\ 1\to if(C_{i}\geq C_{i,\lim}) \end{array}$$

where $f_{0}\;$= 0, $C_{i}$ is the molar concentration of the *i*-th reactive electron acceptor, $C_{i,lim}$ is the limiting molar concentration of the *i*-th reactive electron acceptor, and $\alpha_{i}$ is the dimensionless parameter. Each electron acceptor was assumed to have a limiting concentration ($C_{i,lim}$). When the concentration of an electron acceptor ($C_{i}$) exceeded its limit, the corresponding half-reaction rate was independent of the $C_{i}$ magnitude. Otherwise, the rate was proportional to $C_{i}$ (first-order correlation).

Nitrification reactions are calculated and described using the conventional second-order bimolecular reaction dynamics:

(17) $$r_{NH_{4}^{+}}=k_{NH_{4}^{+}}\cdot C_{NH_{4}^{+}}\cdot C_{O_{2}}$$

Not only do some species act as reactants, but also as products. The net reaction rates of the four species can be expressed as:

(18) $$R_{DOC}=-r_{DOC}$$

(19) $$R_{O_{2}}=-r_{red,1}-2r_{NH_{4}^{+}}$$

(20) $$R_{NO_{3}^{-}}=-r_{red,2}+r_{NH_{4}^{+}}$$

(21) $$R_{NH_{4}^{+}}=-r_{NH_{4}^{+}}$$

In order to facilitate evaluating the nitrogen transformation in the entire porous medium, the mean spatial reaction rate was used in this study. The distribution of the reaction rate of the entire area was integrated, and then divided by the area of the sediment to obtain the mean spatial reaction rate of nitrification ($\bar{r_{NI}}$), the mean spatial reaction rate of denitrification ($\bar{r_{DN}}$), and the mean spatial reaction rate of net denitrification ($\bar{r_{netDN}}$).

(22) $$\bar{r_{NI}}=\frac{1}{A}\int \theta \cdot r_{NH_{4}^{+}}\cdot dA$$

(23) $$\bar{r_{DN}}=\frac{1}{A}\int \theta \cdot r_{red,2}\cdot dA$$

(24) $$\bar{r_{netDN}}=\frac{1}{A}\int \theta \cdot r_{NO_{3}^{-}}\cdot dA$$

If $\bar{r_{netDN}}$ was negative, it indicates that the amount of nitrate consumed by denitrification was greater than that produced by nitrification. Conversely, it indicates that the amount of nitrate consumed by denitrification was less than that produced by nitrification.

#### 2.2.4. Numerical Modeling

In this research, the COMSOL Multiphysics software was used for the simulations [37]. A numerical model of the same scale as the above indoor flume experiment was created. The simulation domain was discretized by a triangular mesh. The mesh was refined along the weir structure considering the research objective and expected flow variation area. The total mesh size was 49,378. The numerical model was built based on the above conceptual model. The transport of non-reactive NaCl was simulated to validate the model. It is assumed that NaCl was completely mixed in surface water so that the concentration of NaCl at the SWI was constant with a value of 40 mol/m^3^. The other parameters used in the model are shown in Table 2.

## 3. Results

### 3.1. Model Validation

The concentration distribution of the NaCl solute in the hyporheic zone is verified by comparing the simulated values of N1 and N2 columns at 30, 60, and 120 min by the model with the measured concentration values in the indoor flume experiment. The N1 and N2 columns are located upstream and downstream from the weir structure respectively, which can better validate the model. To facilitate comparison and verification, the solute concentration is normalized and the dimensionless NaCl concentration $C^{*}$ is obtained by:

(25) $$C^{*}=\frac{C}{C_{0}}$$

where C is the NaCl concentration in the hyporheic zone, and $C_{0}$ is the concentration of evenly mixed NaCl at the surface water–sediment interface. The value of $C^{*}$ ranged from 0 to 1.

**Figure 3:**
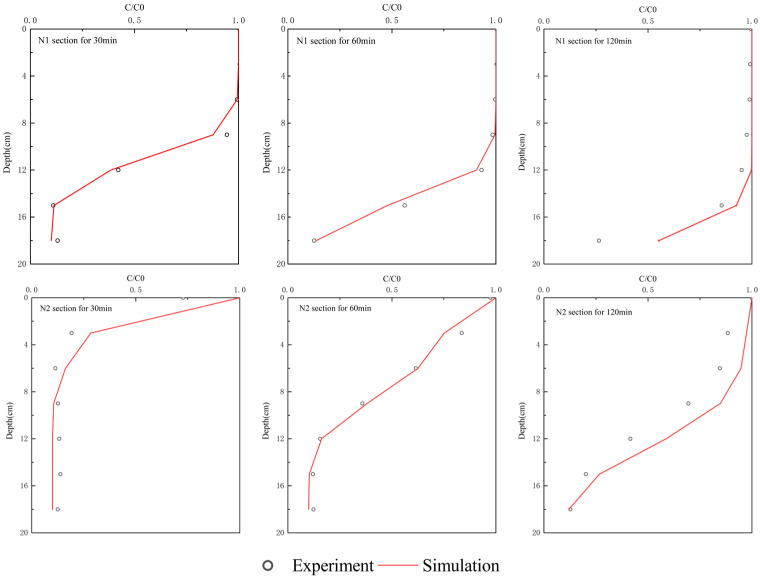
Comparison between the experiment and simulation for NaCl concentrations in different depths and times at N1 and N2 sections.

Evaluation metrics including the root mean square error (RMSE), coefficient of determination (R^2^), and relative error (RE) were used to compare the simulation results with the measured data. Table 3 shows the evaluation metrics and Figure 3 shows the comparison between the measured data and simulated data.

**Figure 4:**
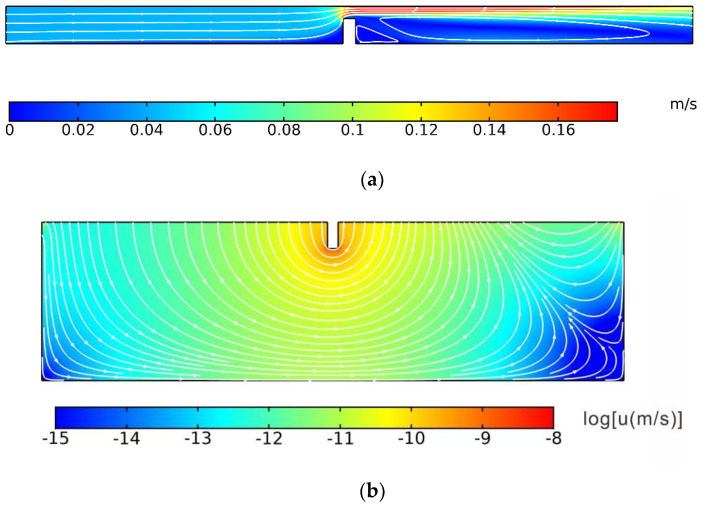
The velocity distribution in (**a**) overlying water and (**b**) porous media.

As the results show, the simulated distribution curves of the NaCl solute concentration for the N1 and N2 column samples were consistent with the measured values in the indoor flume experiment, especially for the short duration. In the flume experiment, the NaCl concentration decreased as the depth increased due to the corresponding small vertical velocity. As time continued, the NaCl concentration became higher due to the continued solute flow into the porous media with the circulation overlying water flow. In comparison with the downstream sampling ports in N2, it is easier for the upstream column to break through the deeper porous medium. It is because the weir alters the pore water flow patterns, and a more rapid downward movement of the solute front occurs upstream of the weir. The RMSE corresponding to the three time points was less than 0.1143, the R^2^ was greater than 0.786, and the RE was between 0.04 and 0.37. It can be seen from the three simulated results that the predicted values of NaCl solute concentration deviate only slightly from the measured values in the indoor flume experiment, which verifies that the numerical modeling method is appropriate to describe the hydrodynamic process and non-reactive solute transport process when a weir structure exists in the hyporheic zone. Moreover, simulated velocity distributions in both the overlying surface water and pore water are shown in Figure 4. The velocity had a sharp increase at the weir structure in the surface water due to the small flow cross-section blocked by the structure. The SWI pressure at the upstream location can be larger than the downstream caused by the total head decrease and the larger surface water velocity at the downstream location. The velocity distribution in the porous medium is shown in Figure 4b. The velocity was relatively small, and the flow direction was from upstream to downstream due to the SWI distribution difference. The maximum velocity occurred around the bottom of the structure, which could reach 2.25 × 10^−4^ m/s.

### 3.2. A hypothetical Nitrogen Transport and Reaction Model

Based on the above-mentioned model, we built a hypothetical model to consider the nitrogen transport and reaction. As mentioned earlier, four species including dissolved oxygen (DO), dissolved organic carbon (DOC), nitrate (NO_3_^−^), and ammonium ion (NH_4_^+^), and three major reactions were simulated. The concentrations of the four representative species were selected by referring to the literature, and are shown in Table 4. It was assumed that the four species were evenly mixed at the SWI and the initial values for the four species in the porous medium were 0 mg/L. The steady-state was also considered.

**Figure 5:**
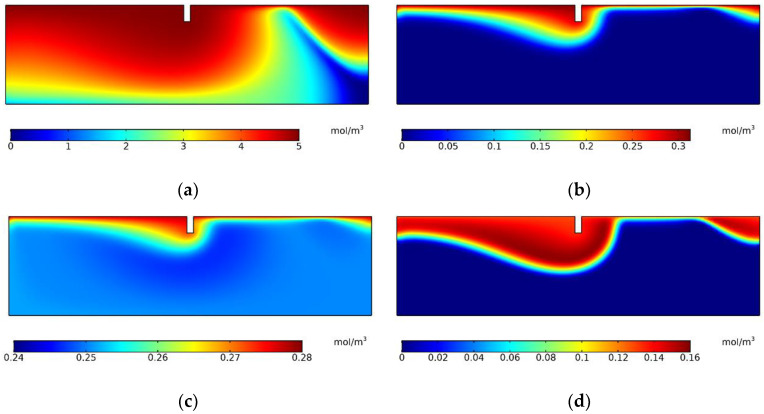
Concentration distributions of the four species: (**a**) DOC; (**b**) DO; (**c**) NH_4_^+^; (**d**) NO_3_^−^.

Figure 5 shows the concentration distributions of the four species. In the numerical model of this study, the limit concentration of DO of 1 mg/L (0.03 mol/m^3^) was defined as the aerobic–anoxic boundary. The concentrations of the four species decreased as the depth increased mainly due to the convective diffusion; however, the concentration boundaries were different because of the reactions. DOC could flow deeper than others because the initial concentration of DOC was larger, and it could satisfy the consumption in the reaction during the entire process. O_2_ controlled the nitrification and denitrification zones. The concentration of O_2_ decreased with the consumption of nitrification and convective diffusion. An aerobic–anoxic boundary occurred as the depth increased. Nitrification mainly occurred above the aerobic–anoxic boundary in the shallow porous zone, while denitrification mainly occurred below the aerobic–anoxic boundary in the deep porous zone. As Figure 5 shows, there was an area with a concentration nearly equal to the initial concentration in the porous medium some distance downstream from the structure, especially for DO, NH_4_^+^, and NO_3_^−^. It can be explained by the velocity distribution in Figure 4b. The species were transported and consumed along the flow lines. If the initial concentration of some species was small, the species may be used up at the end of the long flow path.

## 4. Discussion

As shown above, the weir structure can impact the hydrodynamic process and thus solute transport and reaction. Further, we analyzed some main impact factors by simulating the basic model to enhance the understanding of the structure in ecological restoration engineering in the hyporheic zone.

### 4.1. Effects of Structure Height above the SWI

Pressure distribution along the SWI depends on the height of the weir structure above the porous medium, which can influence the flow and reaction processes in the porous medium. Focusing on the height factors, five height cases were selected including 1, 2, 3, 4 (basic model), and 5 cm, while the other parameters remained unchanged.

As the height increased, the pressure difference at the SWI between upstream and downstream locations increased. It can be concluded that the flow velocity increased with the height in the porous medium. The flow velocity increased from 5.9 × 10^−5^ m/s (Case 1) to 8.93 × 10^−4^ m/s (Case 5). The larger heights can promote the occurrence of hyporheic exchange.

**Figure 6:**
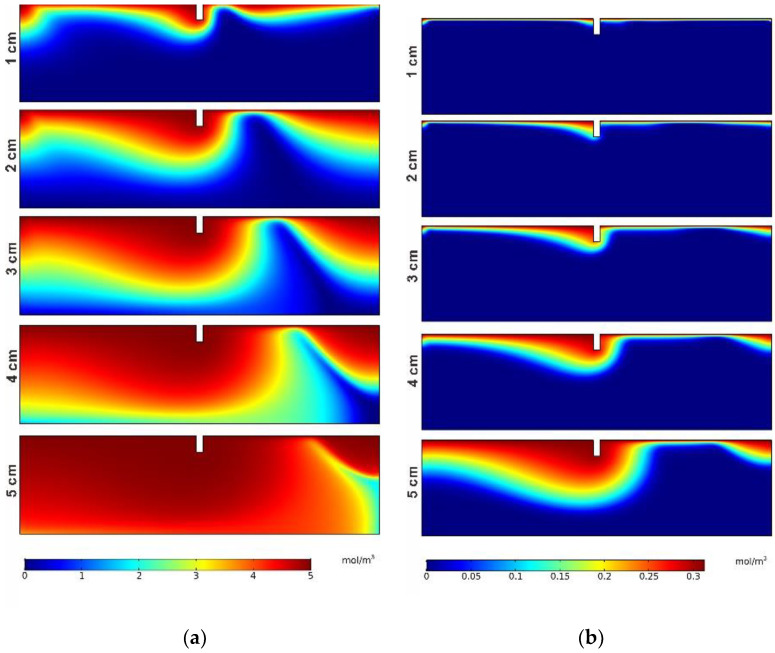

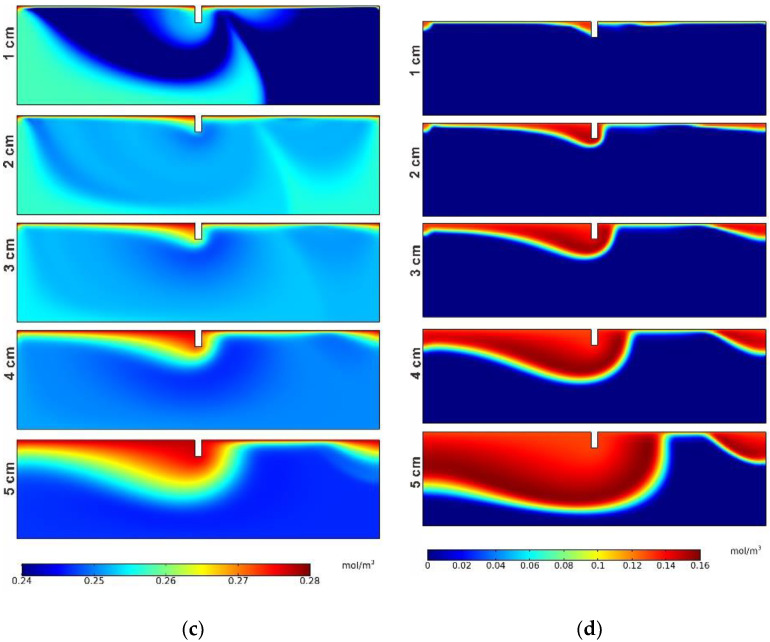
Concentration distributions of the four species in the five different height cases: (**a**) DOC; (**b**) DO; (**c**) NH_4_^+^; (**d**) NO_3_^−^.

The concentration distributions of the four species (DOC, DO, NH_4_^+^, NO_3_^−^) in the five cases are shown in Figure 6. As the surface water entered the hyporheic zone, the DOC solute transported and diffused downward under the five operating conditions. After the concentration reached the maximum value in Figure 6a, it started to decrease continuously. The reason for the concentration of DOC decreasing after reaching the maximum value is that the denitrification reaction with NO_3_^−^ occurred in the anoxia zone. With the height increasing, the concentration front expanded deeper due to the larger flow velocity. The concentration distribution of DO is shown in Figure 6b. The trends of transport and diffusion under the five operating conditions were similar to those of DOC. The diffusion depth of DO at the downstream location was smaller to that at the upstream location. With the increase in structure height, the diffusion depth of DO also increased, but the bottom of the porous medium was not reached. As the DO flow moved downward, nitrification consumed O_2_. The concentration of NH_4_^+^ was illustrated in Figure 6c. The variation in NH_4_^+^ was mainly caused by diffusion and nitrification. Under the condition of H = 1 cm, a ring zone appeared below the structure with a concentration value of about 0.22 mol/m^3^, and the lowest concentration value was about 0.18–0.2 mol/m^3^ near the right boundary. When H = 2, 3, 4, and 5 cm, with the increase in the structure height H, the location of the maximum concentration of NH_4_^+^ migrated downward from the interface, and the NH_4_^+^ concentration decreased from the maximum value to the minimum value of 0.25 mol/m^3^. The NH_4_^+^ front shape was similar to DO due to nitrification. As shown in Figure 6d, the NO_3_^−^ the front became larger as the height increased. The maximum value of the NO_3_^−^ concentration varied from 0.14 to 0.16 mol/m^3^ in the five cases. The concentration of NO_3_^−^ was related to DO and NH_4_^+^ distributions.

**Figure 7:**
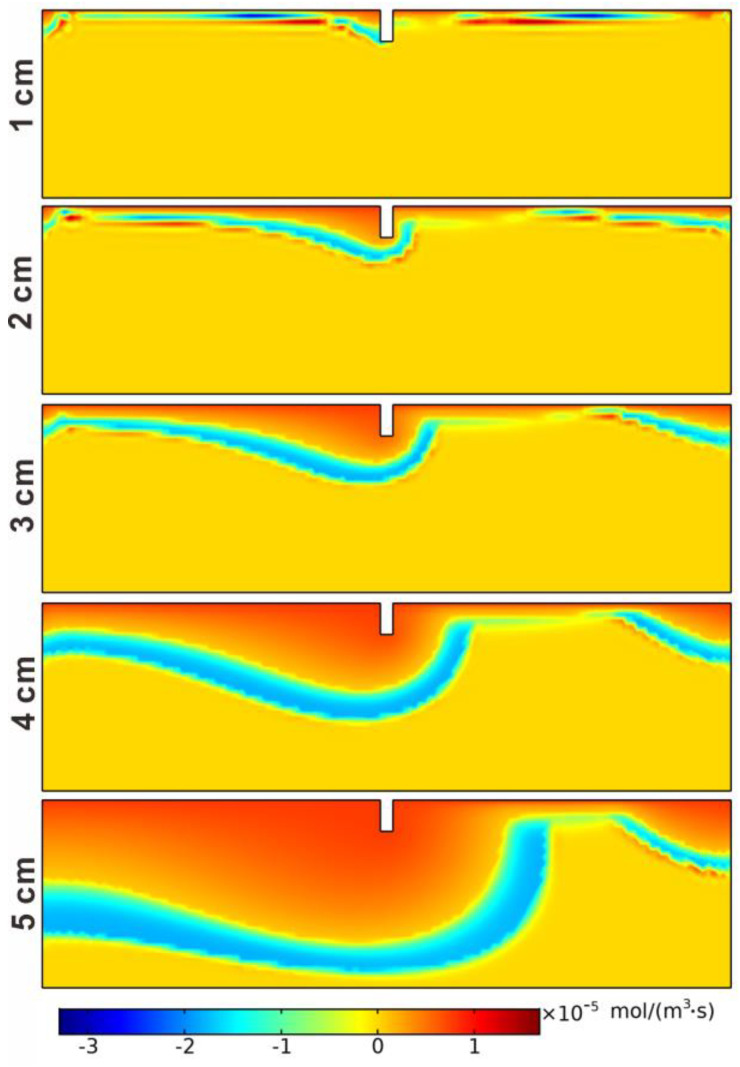
The reaction rates of net denitrification in the five different height cases.

**Figure 8:**
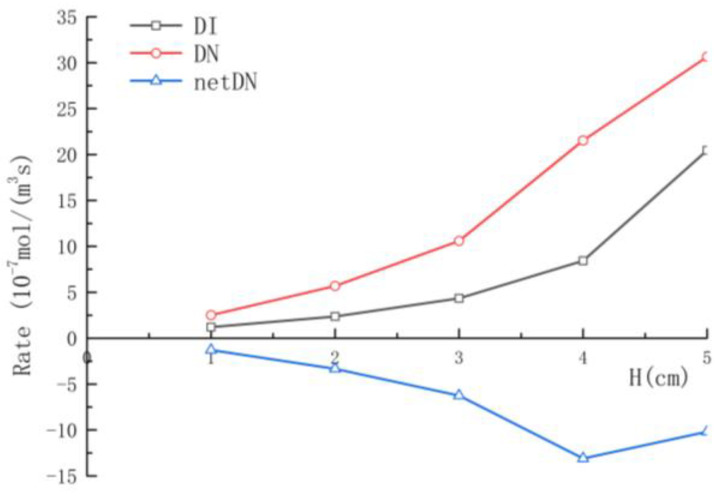
Variations in the mean spatial reaction rates with height.

The reaction rate of net denitrification is demonstrated in Figure 7. Nitrification was dominant in the aerobic region. Because NH_4_^+^ and DO were abundant at the SWI, the nitrification reaction rate had the maximum value near it. With the consumption of the two reactants, the nitrification reaction rate decreased with depth. The denitrification reaction was dominant in the anoxic zone, so the denitrification rate peaked below the aerobic–anoxic boundary and then decreased to 0 mol/(m^3^·s) with depth, forming a narrow but clear denitrification zone. In addition, with the increase in height, the depth of the nitrification zone increased from 0.02 m when H = 1 cm to 0.22 m when H = 5 cm, and the depth of the denitrification zone increased from 0.05 m to 0.28 m. At the same time, the nitrification and denitrification reaction area also increased with the increase in structure height. As shown in Figure 8, the spatial mean rates of nitrification and denitrification also increase with the increase in structure height. The mean rate of the nitrification reaction increased from 1.2126 × 10^−7^ mol/(m^3^·s) to 2.04676 × 10^−6^ mol/(m^3^·s), while the denitrification rate increased from 2.508 × 10^−7^ mol/(m^3^·s) to 3.0646 × 10^−6^ mol/(m^3^·s). The mean net denitrification rate increased as the structure height increased in Case 1–4. Although it decreased in Case 5, it was still larger than in Case 3. It can be seen that the increasing structure height was advantageous to the removal of nitrate in the hyporheic zone and a possible optimal height exists.

### 4.2. Effects of Burial Depth of the Structure

The burial depth of the structure in the porous medium can impact the flow and solute distribution. Five burial depths in the porous medium, 4, 8, 12, 16, and 20 cm, were considered. The height above the SWI was the same as in the basic model and the other parameters remained unchanged.

With the increase in the burial depth of the structure, the maximum value of fluid velocity in the porous medium decreased from 2.47 × 10^−4^ to 1.3 × 10^−4^ m/s. The decrease in velocity was caused by the larger blocking effect of the increased burial depth of the structure in the porous medium.

**Figure 9:**
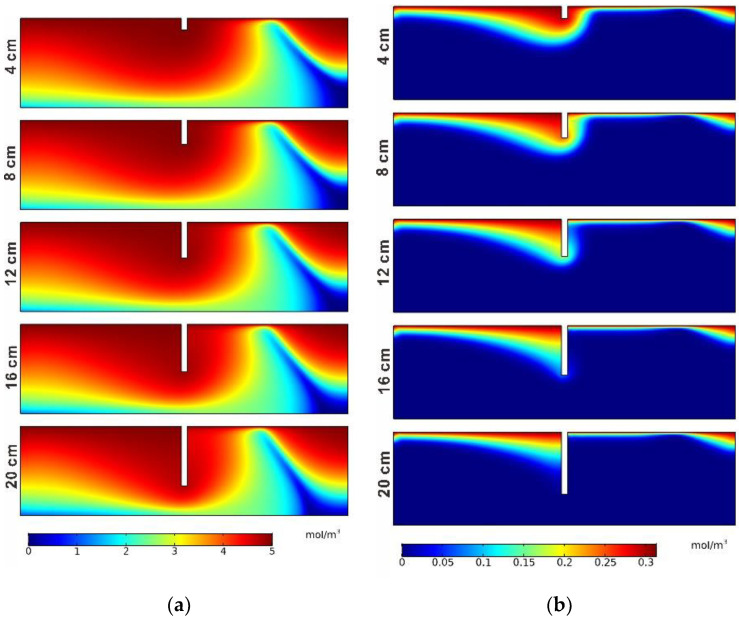

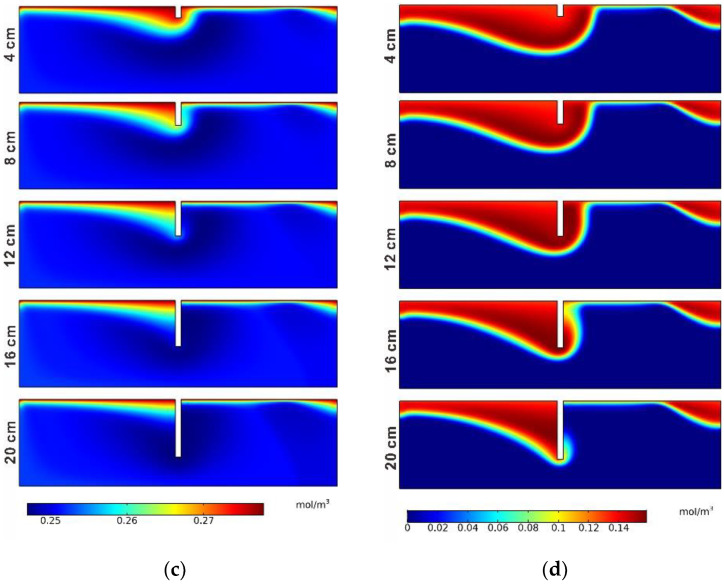
Concentration distributions of the four species in the five different burial depth cases: (**a**) DOC; (**b**) DO; (**c**) NH_4_^+^; (**d**) NO_3_^−^.

The concentration distributions of DOC for different burial depths are shown in Figure 9a. The difference in concentration distributions in different cases was not obvious compared to the cases with different structure heights. In the five cases, the concentration front extended to the bottom of the porous medium. The concentration distributions of DO are shown in Figure 9b. When the burial depth exceeded 12 cm, the concentration diffusion of the DO solute in the porous medium can only occur at the upstream location of the structure. In addition, for the depths of 4, 8, 12, and 16 cm, the diffusion depth of the DO solute was located at 17 cm. However, when the burial depth was 20 cm, the structure depth exceeded the DO solute diffusion maximum depth, and the diffusion depth of the DO solute became shallower and dropped to 13 cm. The concentration distributions of NH_4_^+^ and NO_3_^−^ are shown in Figure 9c,d, respectively. The pattern of variation was similar to that of DO, except for the extension area. With the increase in structure depth, especially when it exceeded the solute peaks of NH_4_^+^ and NO_3_^−^, the solute diffusion only occurred in the upstream region of the structure and the diffusion depth became shallow, while the solute could not diffuse downward in the downstream region. The reason for the changes in the concentrations of the DO, NH_4_^+^, and NO_3_^−^ solutes in the hyporheic zone is that the streamlined distribution in the porous medium was changed with the increase in the burial depth of the structure, which led to the decrease in the concentration value near the structure and the smaller maximum diffusion depth.

**Figure 10:**
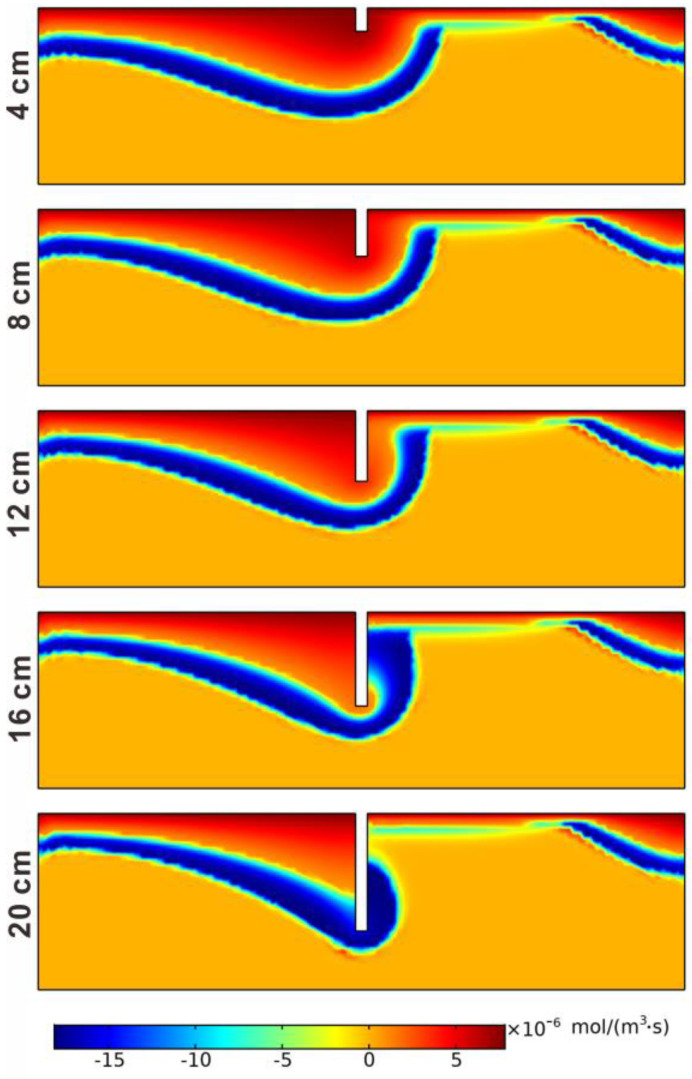
The reaction rates of net denitrification for different burial depths.

**Figure 11:**
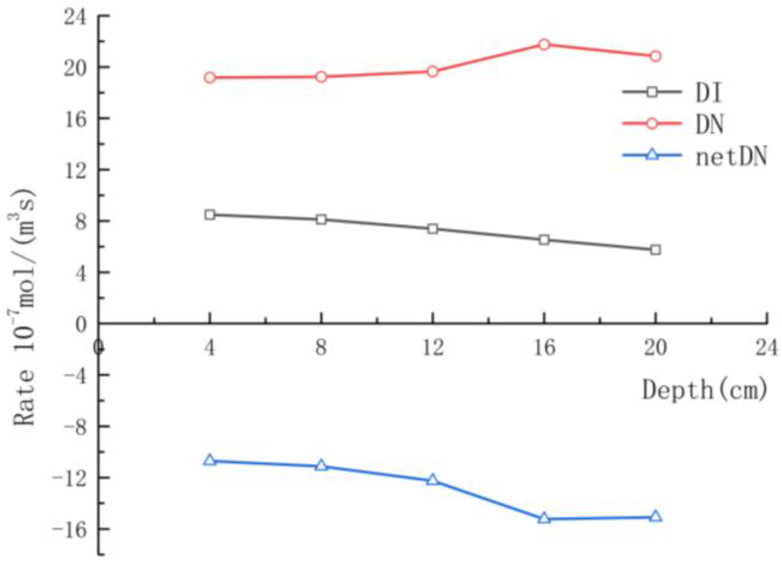
Nitrification, denitrification, and net denitrification rates for different burial depths.

The reaction rates of net denitrification are illustrated in Figure 10. The nitrification reaction area began to change at a burial depth of 12 cm due to the blocking by the structure and the nitrification reaction area in the downstream area becoming smaller. For the depths of 16 and 20 cm, the nitrification reaction area occurred only in the upstream area of the structure, and only a small number of reactions occurred in the downstream area at the surface water–sediment interface. The denitrification reaction area began to change at a burial depth of 16 cm. With the further increase in the burial depth of the structure, when the depth was 20 cm, the structure passed through the reaction area that occurred around the structure. The denitrification area became smaller compared to the other four cases. Figure 11 shows that the spatial mean reaction rate of nitrification decreased with the increase in the burial depth of the structure, from 8.4938 × 10^−7^ to 5.7553 × 10^−7^ mol/(m^3^·s). The denitrification reaction rate increased from 1.9174 × 10^−6^ mol/(m^3^·s) to 2.1769 × 10^−6^ mol/(m^3^·s) with the increase in burial depth. The mean net denitrification rate increased with the structure burial depth increasing, except at a depth of 20 cm. It can be concluded that a larger structure burial depth is beneficial to the removal of nitrate in the hyporheic zone, and a possible optimal depth exists.

### 4.3. Effects of Permeability Characteristics in Porous Media

Permeability characteristics are an important factor for the flow process in porous media, which results in nitrogen transport and transformation. In this section, we focus on the influence of permeability characteristics on the hyporheic zone with a weir structure, which can help guide in selecting the structure located on the target river channel. To this end, different homogeneous porous media are studied.

The effects of different homogeneous permeabilities on nitrogen transport and transformation are studied based on the basic conceptual model. The four permeability coefficient cases are described in Table 5.

**Figure 12:**
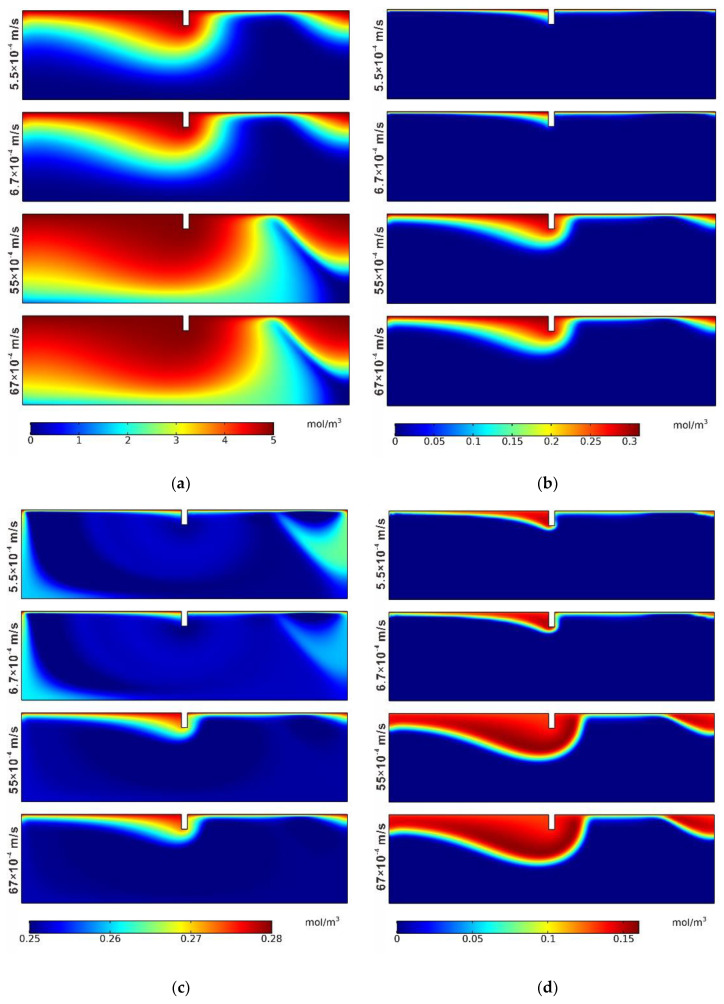
Concentration distributions of the four species for different permeability coefficients: (**a**) DOC; (**b**) DO; (**c**) NH_4_^+^; (**d**) NO_3_^−^.

Figure 12 shows the concentration distributions of the four species for different homogeneous permeability coefficients. With the permeability coefficient increasing, the diffusion depths of DOC, DO, NH_4_^+^, and NO_3_^−^ in the porous media also increased. The reason was that a larger flow velocity in the porous medium was obtained with better permeability characteristics when the pressure difference on the SWI was unchanged in the four cases. The maximum velocity can increase from 1.82 × 10^−5^ m/s to 2.22 × 10^−4^ m/s. As the DO and NH_4_^+^ fronts moved downward, the nitrification areas extended deeper, which enlarged the NO_3_^−^ concentration area and increased the peak concentration.

**Figure 13:**
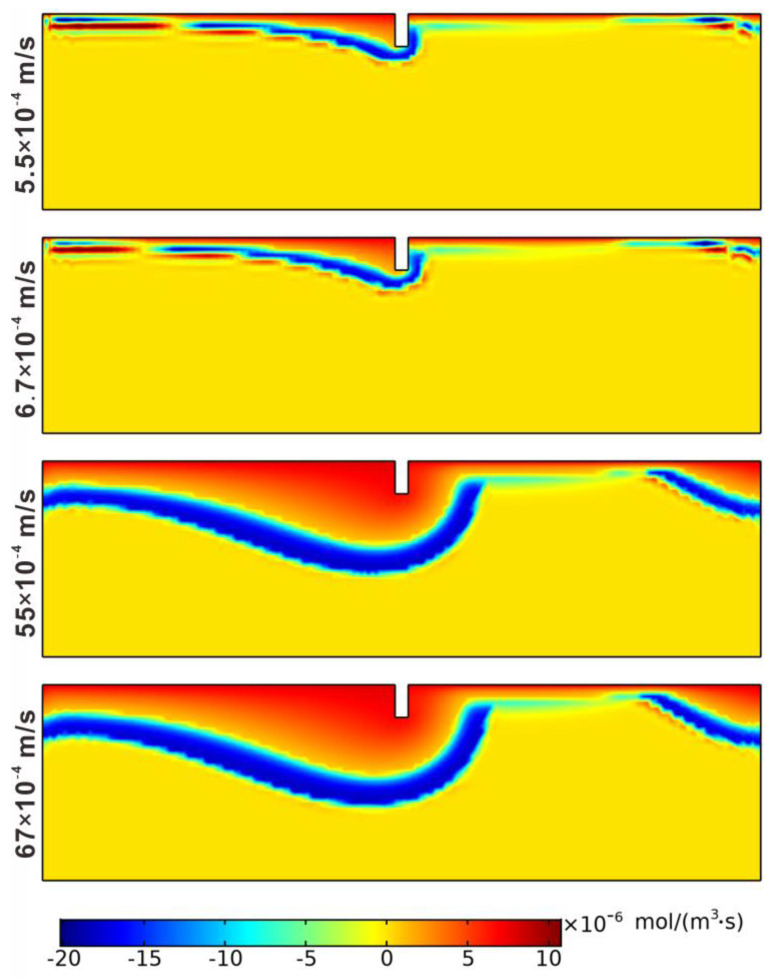
Distributions of reaction rates of net denitrification for different permeability coefficients.

Figure 13 and Table 6 showed the distributions of the reaction rates of net denitrification and the mean spatial reaction rates of nitrification, denitrification, and net denitrification in different cases. With the permeability coefficient increasing, both the nitrification and denitrification reaction areas became larger. The mean spatial reaction rates of nitrification and denitrification increased from 1.8141 × 10^−7^ mol/(m^3^·s) to 8.3481 × 10^−7^ mol/(m^3^·s) and 4.0259 × 10^−7^ mol/(m^3^·s) to 19.0241 × 10^−7^ mol/(m^3^·s), respectively. Better permeability characteristics can promote nitrification and denitrification reactions. However, the mean spatial reaction rate of net denitrification decreases from −2.2118 × 10^−7^ mol/(m^3^·s) to −10.676 × 10^−7^ mol/(m^3^·s), indicating that the denitrification reaction is stronger with the permeability coefficient increasing. It can be concluded that the zone with a larger homogeneous permeability coefficient has the advantage of reducing nitrogen pollution and serves as a better location for the ecological weir structure.

## 5. Conclusions

In this research, nitrogen transport and reaction in the hyporheic zone with an ecological weir structure were analyzed. A non-reactive indoor flume experiment was conducted. The coupled model with the SWI pressure boundary was built, simulated with COMSOL Multiphysics, and validated by experimental data. The hypothetical nitrogen reaction involving three main reaction equations was studied and the important factors of the structure were discussed. The following conclusions can be obtained.

The model coupling surface water and flow in the porous media by the SWI pressure boundary is an appropriate method to describe the flow in the HZ and was verified by the flume experiment. Considering the main nitrogen reactions, the convective diffusion can decline the four species’ concentrations with the depth increasing. The DO and NH_4_^+^ were the main control boundary for nitrification and denitrification. The height of the weir structure above the sediment changed the pressure distribution at the SWI and influenced the velocity field. With the increasing height, the velocity, both in the overlying water and the porous media, can increase, which makes the exchange occur more quickly. The solutes can diffuse much wider and deeper, which results in influencing the entire nitrogen transport and reaction processes. In most cases, increasing the height can promote nitrification and denitrification. The reaction area and concentration front can move deeper with a larger flow velocity. A larger height can better reduce nitrogen pollution. In addition, larger burial depths below the sediment can be also beneficial for nitrogen pollution elimination. The reduced velocity is caused by the larger blocking effect due to the increase in the burial depth of the structure in porous media. However, the rates of nitrification and denitrification are larger, and the net nitrogen elimination effect is better. For future practical engineering, the findings can help optimize the structure design.

Moreover, considering the homogeneous sediment with different permeability coefficients, larger permeability coefficients can accelerate the flow exchange, which results in the species breaking through deeper. DO and NH_4_^+^ can move and diffuse deeper and enhance both the nitrification and denitrification reactions. With the permeability increasing, the effect of nitrogen elimination was promoted. It indicated that the zones with better permeability characteristics are better suited for the weir structure locations.

Furthermore, a few potential research questions related to both the indoor experiment and the coupled model remain to be investigated. First, the scale of the indoor flume may limit the flow and the no-flux boundary can impact the more realistic flow. In the next step, experiments of larger scales or cycle boundaries can be conducted, and realistic nitrogen reactions should be considered to validate the simulation model. Second, more detailed reactions should be considered and simulated in the porous medium. Third, the coupled surface–groundwater model should be improved with more complex ecological structures.

## Figures and Tables

**Table 1:** The reaction equations used in the model.

Reaction Type.	Reaction Equation	Electron Transfer Rate
Aerobic reaction	${\mathrm{CH}}_{2}O+O_{2}\to {\mathrm{CO}}_{2}+H_{2}O$	1
Nitration reaction	${\mathrm{NH}}_{4}^{+}+2O_{2}\to {\mathrm{NO}}_{3}^{-}+2H^{+}+H_{2}O$	-
Denitrification reaction	$5{\mathrm{CH}}_{2}O+4{\mathrm{NO}}_{3}^{-}+4H^{+}\to 5{\mathrm{CO}}_{2}+2N_{2}+7H_{2}O$	0.8

**Table 2:** Parameters used in the numerical model.

Parameter	Water	Sediment
Velocity (m/s)	0.04	-
Density (kg/m^3^)	1000	1680
Kinematic eddy viscosity (Pa·s)	0.001	-
Porosity	-	0.4
Permeability coefficient (m/s)		6.8 × 10^−3^
Fluid diffusion coefficient (m^2^/s)	1 × 10^−10^	-
Longitudinal dispersion (mm)	-	10
Lateral dispersion (mm)	-	1

**Table 3:** Evaluation metrics for different sampling points and periods.

	N1 Column			N2 Column		
Time	*R* ^2^	RMSE	RE (%)	*R* ^2^	RMSE	RE (%)
30 min	0.994	0.0308	0.04	0.707	0.1124	0.37
60 min	0.989	0.0331	0.04	0.988	0.0364	0.06
120 min	0.786	0.1143	0.13	0.896	0.1039	0.15

**Table 4:** The concentrations of the four species.

Species	DOC (mg/L)	DO (mg/L)	NO_3_^−^ (mg/L)	NH_4_^+^ (mg/L)
Value	150	10	8	5

**Table 5:** Permeability coefficients for the four cases.

Case	1	2	3	4
Permeability coefficient (1 × 10^−4^ m/s)	5.5	6.7	55	67

**Table 6:** Mean spatial reaction rates of nitrification, denitrification, and net denitrification.

Case	r_NI_ (1 × 10^−7^ mol/(m^3^·s))	r_DN_ (1 × 10^−7^ mol/(m^3^·s))	r_netDN_ (1 × 10^−7^ mol/(m^3^·s))
1	1.8141	4.0259	−2.2118
2	1.9917	4.5281	−2.5364
3	7.3023	16.9984	−9.6961
4	8.3481	19.0241	−10.676

## Data Availability

The data presented in this study are available on request from the corresponding author. The data are not publicly available due to the subsequent studies will be conducted on this basis.
